# Body Weight Perception and Weight Control Practices among Teenagers

**DOI:** 10.5402/2013/395125

**Published:** 2013-08-18

**Authors:** Darshini Devi Bhurtun, Rajesh Jeewon

**Affiliations:** Department of Health Sciences, Faculty of Science, University of Mauritius, Reduit, Mauritius

## Abstract

*Background*. Weight-loss behaviours are highly prevalent among adolescents, and body weight perception motivates weight control practices. However, little is known about the association of body weight perception, and weight control practices among teenagers in Mauritius. The aim of this study is to investigate the relationships between actual body weight, body weight perception, and weight control practices among teenagers. *Methods*. A questionnaire-based survey was used to collect data on anthropometric measurements, weight perception and weight control practices from a sample of 180 male and female students (90 boys and 90 girls) aged between 13 and 18 years old. *Results*. Based on BMI, 11.7% of students were overweight. Overall, 43.3% of respondents reported trying to lose weight (61.1% girls and 25.6% boys). Weight-loss behaviours were more prevalent among girls. Among the weight-loss teens, 88.5% students perceived themselves as overweight even though only 19.2% were overweight. Reducing fat intake (84.6%), exercising (80.8%), and increasing intake of fruits and vegetables (73.1%) and decreasing intake of sugar (66.7%) were the most commonly reported methods to lose weight. *Conclusion*. Body weight perception was poorly associated with actual weight status. Gender difference was observed in body weight perception.

## 1. Introduction

Despite the increased prevalence of weight concern and weight control practices among teenagers, obesity has been increasing steadily [1, 2]. Females attach much importance on appearance and are preoccupied with their weight from a very young age [3]. They idealise a thin physique. On the other hand, males value a muscular physique, which they often associate with health [4, 5]. To achieve their ideal, teenagers engage in weight control behaviours.

Weight control behaviours are precipitated by body weight perception [5]. Body weight perception refers to the personal evaluation of one's weight as “*underweight*” or “*normal weight*” or “*overweight*” irrespective of actual body mass index [6, 7]. The discrepancy in body weight perception is also known as body image distortion [8]. Teenagers who incorrectly judge their actual body size express a certain degree of body dissatisfaction [9, 10]. Healthy or overweight individuals who perceive themselves as overweight or fat are more likely to engage in weight reduction activities, whereas individuals with an excess body weight who do not perceive themselves overweight will not involve themselves in weight loss behaviours [11, 12]. Teenagers adopt both healthy balanced diet and exercise, but those who are dissatisfied with their body and want to lose weight adopt smoking, use of laxatives, purging, and fasting behaviours [13, 14]. Evidence supports that there is a decline in physical activity levels and consumption of fruits and vegetables and breakfast during adolescence among teenagers who perceive themselves as overweight. 

One's perception does not always reflect reality [15]. Body weight perception is influenced by a number of factors including age, gender, family, peers, media, and ethnicity [16, 17]. Studies reported that even children of eight years old were preoccupied with their body size, and this preoccupation intensified and peaked during adolescence [18, 19]. Females are more inclined to perceive themselves as overweight and engage in undue weight loss practices [12, 20]. While weight reduction is desirable in overweight and obese individual [21], unnecessary weight loss practices have potentially harmful consequences for adolescents including nutritional deficiencies and growth retardation [22, 23]. Teenagers adopt behaviours and attitudes that prevail among their peers, and pressure to be thin has a negative impact on body weight perception [24]. Media constantly depicts images of slender women and muscular men, and this leads to the acceptance of these figures as social norm and may be predictive of body size overestimation or underestimation [25].

Body weight perception helps in understanding and predicting weight control behavior among teenagers [5]. Obesity is becoming a worldwide problem, especially in developing countries like Mauritius. Seven percent of the Mauritian adolescent population is obese and 8.4% is overweight [26]. The prevalence of obesity and chronic diseases is well documented by Ministry of Health and Quality of Life (MoHQL). The Global School-Based Student Health Survey documented health behaviours and protective factors among Mauritian teens [27]. However, little data is available on body weight perception and weight control practices among Mauritian teens. Maintaining a normal body weight is an important element of a healthy life [28]. Given the implications of body weight perception on weight control behaviours, this issue needs to be examined among local teenagers. The aim of the study is to investigate body weight perception among male and female teenagers aged between 13 to 18 years of age using the conceptual framework adapted from Wang et al. [5]. The objectives of the study are to investigate the relationship of actual body weight status and body weight perception among teenagers and to document the impact of body weight perception and weight control practices among teenagers. 

## 2. Materials and Methods

### 2.1. Participants

The study is a questionnaire-based survey carried out among a sample of 180 teenagers (90 boys and 90 girls) aged between 13 to 18 years old. Participants were all Mauritians and healthy (i.e., not suffering from any known diseases). The questionnaires were distributed to participants, and they were requested to complete the questionnaire before undertaking anthropometric measurements. 

Anthropometric measurements included weight and height and were measured as described by Tiwari and Sankhala [29]. The same scale and metre rule were used to measure weight and height, respectively, for all participants. Weight was taken to the nearest 0.5 kg (standard error ±0.5 kg). Height was assessed to nearest 0.01 m (standard error ±0.01 m). BMI was calculated to one decimal place. Each respondent was assigned a weight status based on their age, gender, and calculated BMI score. Weight status was defined as per CDC Growth Chart (BMI < 5th percentile: underweight; BMI ≥ 5th and < 85th percentile: normal weight; BMI ≥ 85th and < 95th percentile: overweight; and BMI ≥ 95th percentile: obese) [30, 31]. However, in this study due to the small number of overweight and obese teenagers, all those having a BMI ≥ 85th percentile were classified as overweight. 

### 2.2. Body Weight Perception and Weight Control Practices

Body weight perception question was adapted from Brener et al. [12], and it was measured with the question “How do you describe yourself?” and the following response set was available: “underweight,” “normal weight,” “overweight,” and “very overweight” or “obese.” For analytical purposes, “overweight” and “very overweight” or “obese” were combined to form the “overweight” response. Weight control referred to any current change or changes made during the past 3 months to eating habits, physical activity, or any other behaviour in an attempt to lose or gain weight. It was assessed with the “yes-no” question, for example, “Are you trying to control your weight?” and “What are you trying to do about your weight?”. “Lose weight” and “Gain weight” were the choices provided to the latter question. Questions and methods on weight control practices were adapted from the studies of Yost et al. [13] and Wardle et al. [32]. The list for weight control practices included exercise; reducing fat consumption; reducing number of snacks eaten in between meal; increasing fruits and vegetable consumption; consuming a balanced diet; reducing the amount of food eaten at meal time; skipping meal and fasting.

### 2.3. Dietary Intake

This measures daily fruit and vegetable consumption and breakfast intake. Whole fruits (apple/pear, orange, kiwi, banana) of different sizes (small, medium, big) and measuring cups were shown to the students to assist them in estimating the amount of fruits and vegetables they ate. The minimum recommended intake of 5 servings of fruits and vegetables as suggested by 2005 Dietary Guidelines for Americans [33] was taken as meeting the recommendation for fruits and vegetables.

### 2.4. Physical Activity

Questions from the Global Health School-Based Survey were used to measure physical activity level [27]. These questions were modified to fit the needs of the current study. Subjects were asked to report the frequency and duration of practice of physical activity. The frequency and duration were multiplied to give the total number of hours of physical activity per week. A student whose total of hours of physical activity per week was 7 hours or more was classified as meeting the recommended level of physical activity per day. 

## 3. Results

### 3.1. Actual Weight Based on BMI

Equal number of boys and girls (90 boys and 90 girls) participated in the study. The average age of the participant was 16 years. Based on body mass index (BMI) (Table 1), 16.6% was underweight (10.0% in boys versus 23.3% in girls); 11.7% were overweight (16.7% in boys versus 4.4% in girls; and the rest of the participants (71.7%) were of normal weight (73.3% in boys versus 70.0% in girls). 

### 3.2. Body Weight Perception

Less than half (42.2%) (Table 1) of the students surveyed correctly perceived their weight. Body weight overestimation was reported by 61.1% of the girls and 14.4% of boys. Among participants with a normal or underweight BMI, 31.7% thought they were overweight. Normal weight and underweight girls had a more inaccurate self-evaluation of their current weight status with 58.3% of them finding themselves to be overweight compared to 10.7% of normal weight and underweight boys. There was substantial gender difference in body weight perception (BWP) (chi-test value = 2.8 × 10^−7^). 

### 3.3. Weight Control Practices

Half of the surveyed population were engaged in weight control practices: 43.3% of the respondents reported trying to lose weight while 6.7% reported that they were engaged in weight gain activities. More than twice as many girls participants (61.1%) were involved in weight loss practices compared to boys (25.6%). Based on BMI (Table 2), 80.8% of those who were involved in weight loss behaviours had a BMI < 85th percentile (47.8% boys versus 94.5% girls). There was a considerable correlation between body weight perception and weight control activities such that 88.5% of those involved in weight loss behaviours overestimate their body weight. Body weight perception is a strong determinant of weight control practices (chi test value—3.1 × 10^−44^). The result of the study showed that “appearance” and “health” were the two most cited weight concerns (55.6% respondents reported “appearance” and 43.3% reported “health”). No gender difference was observed in weight concern (chi-test value = 0.17).

### 3.4. Types of Weight Control Practices

Ten different dieting practices were assessed to determine weight loss practices. The most commonly cited methods by all the respondents were reducing fat consumption (84.7%); exercise (80.8%); increasing fruit and vegetable consumption (66.7%); reducing the amount of food eaten at meal times (59.0%); reducing the quantity of snacks consumed between meals (48.8%) and having a balanced diet (32.1%). Skipping meal (11.5%) and fasting (5.1%) was of a low frequency. However, only 4.8% of those who reported having recourse to exercise to lose weight were achieving the recommended daily one hour of physical activity; and 3.5% of those who reported increasing fruits and vegetables were actually meeting the minimum 3 servings of vegetables and 2 servings of fruits per day. No gender difference was observed in the type of weight loss and the use of healthy and unhealthy methods to lose weight. Teens received information on weight control from different sources. The current study reveals that media (62%) was the major source of information for this purpose followed by parents (24%) and peers (14%). 

### 3.5. Trends in Physical Activity, Breakfast Consumption, and Fruit and Vegetable Consumption among Weight Loss Teenagers

Physical activity level and eating habits were compared by reported attempt to weight loss. No differences in physical activity level and eating habits were observed. Only 3.8% of the respondents (8.7% in boys versus 1.8% in girls) who reported trying to lose weight were meeting the daily one hour of physical activity. Slightly over half (59.4%) of the sample were having breakfast daily. However, among them, only 19.6% included a fruit in their breakfast. Results of the study show that in general intake of fruits and vegetables was low. A meagre 1.7% of the population sampled were consuming 5 servings of fruits and vegetables every day. Interestingly, 70.9% girls on weight loss did not eat any fruit daily. 

## 4. Discussion

The purpose of the study was to investigate body weight perception and weight control practices among teenagers, and the outcome of the study supports the general trend that exists. Actual weight status was poorly correlated with weight perception. Gender differences were observed in body weight perception and weight control practice. Many more females overestimated their body weight and were engaged in weight loss activities than males.

Studies demonstrated that body weight perception tends to be inaccurate when compared to BMI [12, 13]. A similar trend was found among Mauritian teenagers. Marked differences were observed between BMI and weight perception. Forty-two percent of the participants inaccurately perceived their weight. Consistent with other studies [34], the outcome of this survey indicated that body weight exaggeration was more prevalent in girls than boys. Boutelle et al. [35] also documented that in general teenagers were more likely trying to lose weight. The outcome of the survey indicated a similar trend. Among the students who were involved in weight control, 86.7% of students reported trying to lose weight. Weight loss behaviours are more pronounced among females [1]. The current findings support this pattern. The prevalence of weight-loss practices was higher among girls (61.1% girls versus 25.6% boys). 

Studies reported that body weight perception is a strong determinant factor in weight control rather than actual BMI [3, 5, 7, 8, 36]. The findings of this study corroborate with earlier reports. A large proportion (88.5%) of teenagers engaged in weight reduction activities perceived themselves as overweight even though only 19.2% of them actually have a BMI above the 85th percentile on the growth chart. Research evidence support that female adolescents are more likely to diet and engage in unhealthy weight-loss behaviours more frequently than males [7]. However, in the current study, both male and female teenagers were equally likely to use food manipulation and adopt healthy and unhealthy methods for weight control. Lowry et al. [1] documented that high school adolescents exercised and reduced intake of fat and ate fruits and vegetables to lose weight. Identical behaviours were observed among the weight-loss teenagers interviewed. The 3 most commonly adopted behaviours to lose weight were reduction of intake of fat (84.6%), exercise (80.8%), and consuming fruits and vegetables (66.7%). Reducing amount of food eaten at meal times was reported by more than half of the boys and girls (52.2% boys and 61.8% boys). Skipping meal and fasting as weight loss strategies were reported by 11.5% and 5.1%, respectively, and these numbers are lower than those reported by Lowry et al. [1]. 

Malinauskas et al. [3] and Wang et al. [5] documented that those adolescents who reported trying to lose weight did not have higher physical activity level or fruit and vegetable intake than others. Although the aim of the study was not to assess the association between individual's reported tendencies of weight loss and practices as well as physical activity level and fruits and vegetables intake, it was observed that those who reported exercising were relatively inactive and were not meeting the international recommendations for fruits and vegetables, respectively. This is, agreement with Nowak [37] who reported that there is no difference in exercise frequency between Australian adolescents who were trying to lose weight and those who were not. 

A decline in physical activity is noted among teenagers worldwide [38]. Results herein indicate a similar pattern with an overall response rate of 6.1% meeting the international recommendations for physical activity. There was no difference in physical activity level between weight-loss and nonweight-loss students. Four percent of weight-loss teens were active for one hour daily. In fact, 14.5% of weight loss girls did not exercise at all, and this is possible due to body dissatisfaction that hinders participation in physical activity [39, 40]. The majority of weight loss teens (95.8%) expressed some body dissatisfaction in that they perceived themselves overweight, and this could partly explain their poor engagement in physical activity. Similar to other adolescents' population [5, 41], the students in the current study had low fruit and vegetable intake. There was no difference in consumption pattern of fruits and vegetables between those who reported trying to lose weight and others. Only 2.6% of the weight-loss students met the minimum recommended 5 servings of fruit and vegetable per day. Statistics in the US showed that 11–37% of school children and adolescents did not have breakfast regularly [42]. The current study demonstrated a similar scenario as no difference was observed in breakfast consumption between weight-loss and nonweight-loss adolescents. 

## 5. Conclusion and Limitations

The findings of the study demonstrated that body weight perception was poorly associated with BMI among Mauritian teenagers. Gender difference was observed in body weight perception with many more female adolescents overestimating their body size. Teenagers who perceived themselves as overweight engaged in weight reducing activities and exercise and reducing fat intake which were the two most commonly reported methods for weightloss in both genders. In light of the high prevalence of body weight misperception existing among the teenagers and the fact that body weight perception motivated weight control behaviours, promotion of healthy body image perception and healthy eating by schools and health organisations is important. Since media holds an important place in influencing body weight perception, media can be used to vehicle healthful messages. 

One major limitation in this study is sample size which may not be representative of all teenagers. In addition, socioeconomic status (SES) of the participants was not considered. Future studies should consider the latter as it influences body image and weight control practices [43]. 

## Figures and Tables

**Table 1:** Body weight based on BMI and body weight perception.

	Weight based on BMI	Self-judged status		
		Underweight	Normal weight	Overweight
All (*n* = 180)	Underweight	12	11	7
	Normal weight	2	77	50
	Overweight	0	6	15

Boys (*n* = 90)	Underweight	4	5	0
	Normal weight	2	56	8
	Overweight	0	4	11

Girls (*n* = 90)	Underweight	8	6	7
	Normal weight	0	21	42
	Overweight	0	2	4

**Table 2:** Weight control practices based on BMI.

	BMI	No weight control	Weight loss	Weight gain	Total
All (*n* = 180)	Underweight	14	7	9	30
	Normal weight	70	56	3	129
	Overweight	6	15	0	21

Boys (*n* = 90)	Underweight	4	0	5	9
	Normal weight	52	11	3	66
	Overweight	3	12	0	15

Girls (*n* = 90)	Underweight	10	7	4	21
	Normal weight	18	45	0	63
	Overweight	3	3	0	6
